# Anle138b Partly Ameliorates Motor Deficits Despite Failure of Neuroprotection in a Model of Advanced Multiple System Atrophy

**DOI:** 10.3389/fnins.2016.00099

**Published:** 2016-03-10

**Authors:** Lisa Fellner, Daniela Kuzdas-Wood, Johannes Levin, Sergey Ryazanov, Andrei Leonov, Christian Griesinger, Armin Giese, Gregor K. Wenning, Nadia Stefanova

**Affiliations:** ^1^Division of Neurobiology, Department of Neurology, Medical University InnsbruckInnsbruck, Austria; ^2^Neurologische Klinik, Klinikum der Ludwig-Maximilians-Universität MünchenMunich, Germany; ^3^NMR based structural Biology, Max Planck Institute for Biophysical ChemistryGöttingen, Germany; ^4^DFG Center for Nanoscale Microscopy and Molecular Physiology of the BrainGöttingen, Germany; ^5^Zentrum für Neuropathologie und Prionforschung, Ludwig-Maximilians-Universität MünchenMunich, Germany

**Keywords:** Anle138b, α-synuclein, multiple system atrophy, glial cytoplasmic inclusions, 3- nitropropionic acid

## Abstract

The neurodegenerative disorder multiple system atrophy (MSA) is characterized by autonomic failure, cerebellar ataxia and parkinsonism in any combination associated with predominantly oligodendroglial α-synuclein (α-syn) aggregates (glial cytoplasmic inclusions = GCIs). To date, there is no effective disease modifying therapy. Previous experiments have shown that the aggregation inhibitor anle138b reduces neurodegeneration, as well as behavioral deficits in both transgenic and toxin mouse models of Parkinson's disease (PD). Here we analyzed whether anle138b improves motor skills and reduces neuronal loss, as well as oligodendroglial α-syn aggregation in the PLP-α-syn transgenic mouse challenged with the mitochondrial toxin 3-nitropropionic acid (3-NP) to model full-blown MSA. Following 1 month of treatment with anle138b, MSA mice showed signs of motor improvement affecting stride length, but not pole, grip strength, and beam test performance. Loss of dopaminergic nigral neurons and Purkinje cells was not attenuated and GCI density remained unchanged. These data suggest that the pathology in transgenic PLP-α-syn mice receiving 3-NP might be too advanced to detect significant effects of anle138b treatment on neuronal loss and intracytoplasmic α-syn inclusion bodies. However, the partial motor amelioration may indicate potential efficacy of anle138b treatment that may be mediated by its actions on α-syn oligomers or may reflect improvement of neuronal dysfunction in neural at risk populations. Further studies are required to address the efficacy of anle138b in transgenic α-syn models of early-stage MSA and in the absence of additional toxin application.

## Introduction

Multiple system atrophy (MSA) is a progressive, adult-onset neurodegenerative disorder that belongs to the spectrum of α-synucleinopathies (ASP). MSA patients present with parkinsonism, cerebellar, autonomic, and pyramidal dysfunction in any combination (Fanciulli and Wenning, 2015). Currently, no treatment options are available to stop the progression of this fatal disease, only the amelioration of symptoms is feasible (Eschlbock et al., 2016). Two major types of MSA are distinguished: striatonigral degeneration (SND) underlies the parkinsonian variant of MSA whereas olivopontocerebellar atrophy (OPCA) is associated with the cerebellar subtype. In end-stage disease usually both SND and OPCA are present. In MSA α-synuclein (α-syn) accumulates in oligodendrocytes (glial cytoplasmic inclusions = GCIs or Papp-Lantos bodies). However, α-syn-positive inclusions may appear in neurons (Cykowski et al., 2015) as well and sometimes also in astroglial cells similar to other ASP, such as Parkinson's disease (PD) and dementia with Lewy bodies (DLB). The various inclusions appear to contribute to the neurodegeneration associated with MSA (Wenning et al., 2008). More recent evidence suggests that toxic α-syn aggregates might induce a prion-like spreading in MSA and related ASP (Angot et al., 2010; Reyes et al., 2014; Prusiner et al., 2015).

In previous work we have replicated MSA-like glial inclusions and mild SND in mice overexpressing human α-syn (hα-syn) under the control of the proteolipid protein (PLP) promoter (Kahle et al., 2002; Stefanova et al., 2005; Fellner et al., 2015; Stefanova and Wenning, 2015). In this PLP-α-syn mouse model, additional chronic oxidative stress induced by intraperitoneal (IP) 3-nitropropionic acid (3-NP) injections led to full-blown MSA-like pathology including widespread insoluble α-syn aggregates in oligodendroglia associated with SND and OPCA, as well as profound microglial and astroglial activation. Moreover, the oxidative stress in PLP-α-syn mice resulted in enhanced motor deficits (Stefanova et al., 2005; Stefanova and Wenning, 2015). Because of the subacute time course and because of the similarities of pathological and behavioral deficits reported in the PLP-α-syn mice treated with 3-NP and human clinically overt MSA, this mouse model provides a useful test bed to investigate new therapeutic drugs and to transfer positive candidates to clinical trials (Kuzdas-Wood et al., 2014). Therefore, we sought to investigate the disease-modifying properties of the aggregation inhibitor, anle138b, in PLP-α-syn mice treated with 3-NP. In a recent study anle138b was shown to bind structure-dependently to pathological aggregates and furthermore to inhibit the formation of pathological oligomers, including α-syn, *in vitro* and *in vivo* (Wagner et al., 2013, 2015). Moreover, Wagner and colleagues reported an inhibition of aggregate formation, neuronal degeneration and disease progression in different mouse models of PD without detectable toxicity at therapeutic doses (Wagner et al., 2013; Levin et al., 2014). For the first time we here investigate the effects of anle138b in a MSA mouse model combining overexpression of α-syn in oligodendrocytes and chronic oxidative stress caused by 3-NP injections.

## Materials and methods

### Animals and treatments

All animal experiments were performed according to ethical guidelines and Austrian law as well as with permission from the Federal Ministry of Science and Research, Austria. All efforts were made to reduce the number of animals used and minimize their suffering. All mice were bred and maintained under temperature-controlled, pathogen free conditions, and 12-h light/dark cycle granting free access to water and food at the Animal Facility of Medical University Innsbruck. In the present study a total of 28 homozygous transgenic PLP-α-syn mice, obtained from Prof. Philipp Kahle (Tübingen, Germany) and described previously (Kahle et al., 2001), were used. All mice were genotyped applying tail clip and PCR for hα-syn using following primers with a product size of 450 bp: fwd: 5′-ATG GAT GTA TTC ATG AAA GG-3′; rev: 5′-TTA GGC TTC AGG TTC GTA G-3′. Determination of oligodendroglial AS overexpression was previously reported in the PLP-α-syn mice using immunofluorescence (Kahle et al., 2001; Stemberger et al., 2010).

Twelve month old homozygous transgenic PLP-α-syn mice were randomized into two treatment groups receiving either vehicle or aggregation inhibitor anle138b [3-(1,3-benzodioxol-5-yl)-5-(3-bromophenyl)-1H-pyrazole] treatment. The anle138b group started receiving food pellets (Ssniff, Soest, Germany) that contained the compound anle138b (2 g compound/1 kg food) whereas the control group received food pellets without the compound anle138b 1 week prior to 3-NP intoxication. Body weight of the mice was controlled daily. Food pellets were provided throughout the whole experiment. After 1 week both groups were intoxicated with 3-NP to induce the full-blown pathology similar to human disease (Stefanova et al., 2005). The 3-NP IP treatment was accomplished using following scheme as described previously: 4 × 10, 4 × 20, 4 × 40, 4 × 50 mg/kg (injection volume 200 μL; Stefanova et al., 2005). 3-NP was dissolved in saline and pH was adjusted to 7.4 using 1 mol/L sodiumhydroxid (NaOH). During the intoxication period IP injections of 3-NP were conducted every 12 h. From day 5 of the intoxication period till the end of the experiment parallel to the delivery of anle138b with the food, mice received 250 mg/kg b. w. anle138b by oral gavage (Unimed, Switzerland) twice a day. This was done to avoid any decrease in anle138b dosing due to disability and reduced food intake after the 3-NP intoxication. Control mice received vehicle by oral gavage according to the same time schedule. Behavioral assessment was performed following oral treatment starting with day 22 and mice were sacrificed 4 weeks after starting treatment with food pellets according to the following protocols.

### Behavioral tests

All behavioral tests were performed by a researcher who was blinded to the treatment status of the animals.

#### Standardized motor behavioral scale

Motor score analysis to assess the severity of 3-NP induced motor disability of the treated transgenic PLP-α-syn mice was performed every day beginning with the first IP 3-NP injection. To estimate hindlimb clasping, general locomotor activity, hindlimb dystonia, truncal dystonia, and postural challenge response a previously described rating scale was applied (0, normal; 1, slightly disturbed; 2, markedly disabled; Fernagut et al., 2002; Stefanova et al., 2005). For each motor score analysis per day a total score was calculated.

#### Open field activity

The Flex Field Activity System (San Diego Instruments, USA) was used to assess the locomotor activity of the mice upon treatment as previously described (Stefanova et al., 2012). Thereby, 544 photo-beam channels monitor and count horizontal and vertical locomotor activity of the mice that were placed in the center of the open field (40.5 × 40.5 × 36.5 cm) for a period of 15 min. The tests were performed in a dark room isolated from external noises and light during the test period. The total counts in the horizontal and vertical (rearing) activity were further analyzed.

#### Stride length analysis

To test the stride length of the mice after treatment the DigiGait™Analysis System (Mouse Specifics, USA) was used as described elsewhere (Amende et al., 2005; Stefanova et al., 2012). Briefly, mice were put on motor-driven transparent treadmill belt with a high-speed digital video camera placed below the belt which captured the gait of the mice (speed 25 cm/s). The specific DigiGait Software 9.0 (Mouse Specific, USA) was used to analyze the collected images and evaluate the mean stride length of each forelimb and hindlimb of the mice.

#### Pole test

The pole test has been described as a helpful behavioral test for evaluating movement disorders caused by striatal dopamine depletion (Matsuura et al., 1997; Fernagut et al., 2002). Each mouse was trained and accustomed to the test 1 day before. For the test a wooden vertical pole with rough surface, 1 cm wide and 50 cm high was used. The mice were placed with the head up at the top of the pole and the time for turning downwards (*T*_turn_) and the total time for climbing down the pole until the mice reached the floor with all four paws (*T*_total_) was measured in five attempts. For statistical analysis only the best performance of the five trials was used (Fernagut et al., 2002). For mice that were unable to successfully complete the test a default value of 120 s was taken into account (Stefanova et al., 2005).

#### Beam walking test

Fine motor coordination and balance capacities of the mouse were assessed employing the beam walking test (Luong et al., 2011). Thereby, the mice have to traverse 50 cm of length on a horizontal wooden beam (width 0.9 cm). The day before the test mice were trained to get accustomed to the task. The time for traversing the beam and the number of slips were evaluated in five consecutive attempts. Again the best performance of the five trials of each mouse was used for statistical analysis.

#### Grip strength analysis

To measure forelimb grip strength of mice upon 3-NP injection, the ability to remain clinging to an inverted wire grid for up to 1 min under constant mild shaking was tested in three trials. The best performance was used for statistical analysis.

### Tissue processing

On day 28 after starting the anle138b treatment, animals were perfused under deep thiopental anesthesia (12 mg/100 g body weight, IP) using phosphate buffered saline (PBS; 25 mM, 0.9% NaCl, pH 7.4) followed by 4% paraformaldehyde (pH 7.4, Sigma-Aldrich, Austria). After removing brains quickly, they were post-fixed in 4% PFA over night at 4°C, and then kept in 30% sucrose (Sigma-Aldrich, Austria) solution until they sank at 4°C. Afterwards, brains were frozen using 2-methylbutan and stored at -80°C until further processing. Whole brains were cut on a freezing microtome (Leica, Nussloch, Germany) processing 40 μm thick sections, whereby one section was collected on slides and six sections were kept free-floating and either dried or kept in assorter buffer.

### Immunohistochemistry for light microscopy

Immunohistochemical staining was performed according to a standard immunoperoxidase protocol for free-floating sections using following antibodies: mouse anti-dopamine- and cAMP-regulated phosphoprotein (DARPP-32; 1:5000; BD Biosciences, USA), mouse monoclonal anti-tyrosine hydroxylase (TH; 1:1000; Sigma, USA), rat anti-human α-syn (aa 116-131 hα-syn; dilution and company please; 15G7, Enzo Life Sciences, Germany), rabbit anti-phosphorylated α-syn (pα-syn; 1:1000; Abcam, UK), and mouse anti-nitrated α-syn (nα-syn; 1:1000; Invitrogen, Zymed Laboratories, USA). In case of the visualization of partially proteinase-K (PK)-resistant human α-syn aggregates, tissue slices were pre-stained with haematoxylin and digested with PK before the incubation with antibodies against α-syn (Neumann et al., 2004). Biotinylated horse anti-mouse IgG, biotinylated goat anti-rat IgG, and biotinylated goat anti-rabbit IgG (1:200; Vector Laboratories, USA) were used as secondary antibodies. Vectastain ABC reagent (Vector Laboratories, USA) and 3,3′-diaminobenzidine were applied to visualize the immunohistochemical binding sites. Stained sections were mounted onto gelatin-coated slides, dehydrated and coverslipped with Entellan.

### Image analyses

Stereological analysis was performed by an unbiased investigator at the Nikon E-800 microscope equipped with Nikon digital camera DXM 1200 and Stereoinvestigator Software (MicroBrightField Europe e.K., Germany) as described previously (Mayhew and Gundersen, 1996; Stefanova et al., 2012). For the estimation of the number of dopaminergic neurons in substantia nigra pars compacta (SNc) the optical fractionator was applied. The number of DARPP-32-stained Purkinje cells was estimated in a region outlined to include only the Purkinje cell layer, and the number of PK-resistant GCIs per mm^2^ was estimated in the SNc, SN reticulata (SNr), stratum purkinjense, and stratum granulosum of the cerebellar cortex (German et al., 2001; Stefanova et al., 2012). Furthermore, the percentage of nα-syn- and pα-syn-positive inclusions was analyzed by determining mean gray values of the nα-syn and pα-syn immunoreactivity in the same regions as mentioned for the estimation of the PK-resistant GCIs at constant acquisition camera settings with values of 0 for black and 256 for white. The optical density was measured after the transformation of mean gray values by using the formula OD = −log (mean gray/256) as previously described (Stefanova et al., 2012).

### Statistical analyses

All statistical analyses were conducted using the software Graph-Pad Prism 5 (Graphpad Software, San Diego, CA). The mean ± S.E.M was used to present the results. Unpaired *t*-test was used for the comparison of the behavioral performance of the two groups, if not indicated otherwise. A *P*-value < 0.05 was considered statistically significant.

## Results

### Anle138b treatment induces mild motor improvement in the PLP-α-syn mice challenged with 3-NP

**Figure 1:**
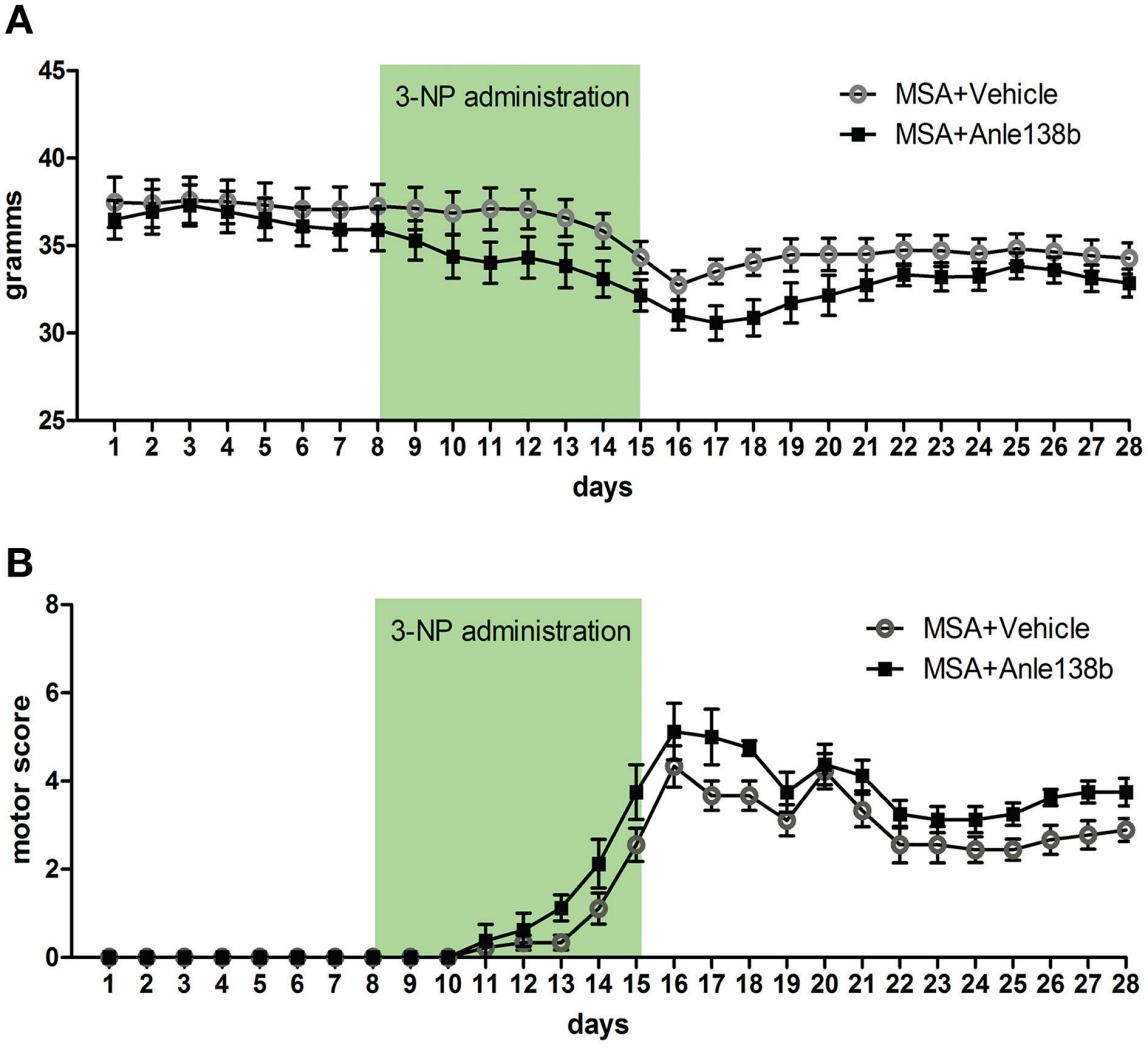
**Effects of anle138b on body weight and motor score**. **(A)** Body weight was comparable in anle138b- and vehicle-treated PLP-α-syn mice. A decrease of body weight was observed in both groups upon intraperitoneal 3-NP injections (day 8–15), yet body weight increased after 3-NP injections again and remained steady. **(B)** Motor score evaluation of the treated animals was performed daily. Thereby, for the evaluation of hindlimb clasping, general locomotor activity, hindlimb dystonia, truncal dystonia, and postural challenge response a previously described rating scale was applied (0, normal; 1, slight disturbed; 2, markedly disabled). The total motor score per day increased permanently with highest scores on day 16. Termination of 3-NP injections led to a slight improvement of the motor score in both treatment groups. No significant differences were detected between mice treated with vehicle or anle138b. Data are presented as mean ± SEM. PLP-α-syn mice treated with vehicle *n* = 10, treated with anle138b *n* = 8. Data were analyzed by two-way repeated measures ANOVA with *post-hoc* Bonferroni's test.

Body weight of vehicle and anle138b treated PLP-α-syn mice was monitored throughout the experiment. No significant differences in body weight between the groups were detected throughout the study. Upon IP injections of 3-NP a typical reduction of body weight was measured with the lowest recordings at the end of 3-NP administration. After day 16 a mild increase followed by a stabilization of the body weight was observed (Figure 1A). Moreover, starting with 3-NP administration the motor score of vehicle- and anle138b-treated mice was assessed using the Standardized Motor Behavioral Scale described by Fernagut et al. (2002). As expected the motor score increased in both groups upon 3-NP injections with the highest scores at the end of 3-NP administration. The termination of 3-NP injections was followed by slight improvement of the motor score that stayed stable until the end of the experiment. We did not observe any statistically significant difference in the motor score at any time point regarding the two treatment groups (Figure 1B).

**Figure 2:**
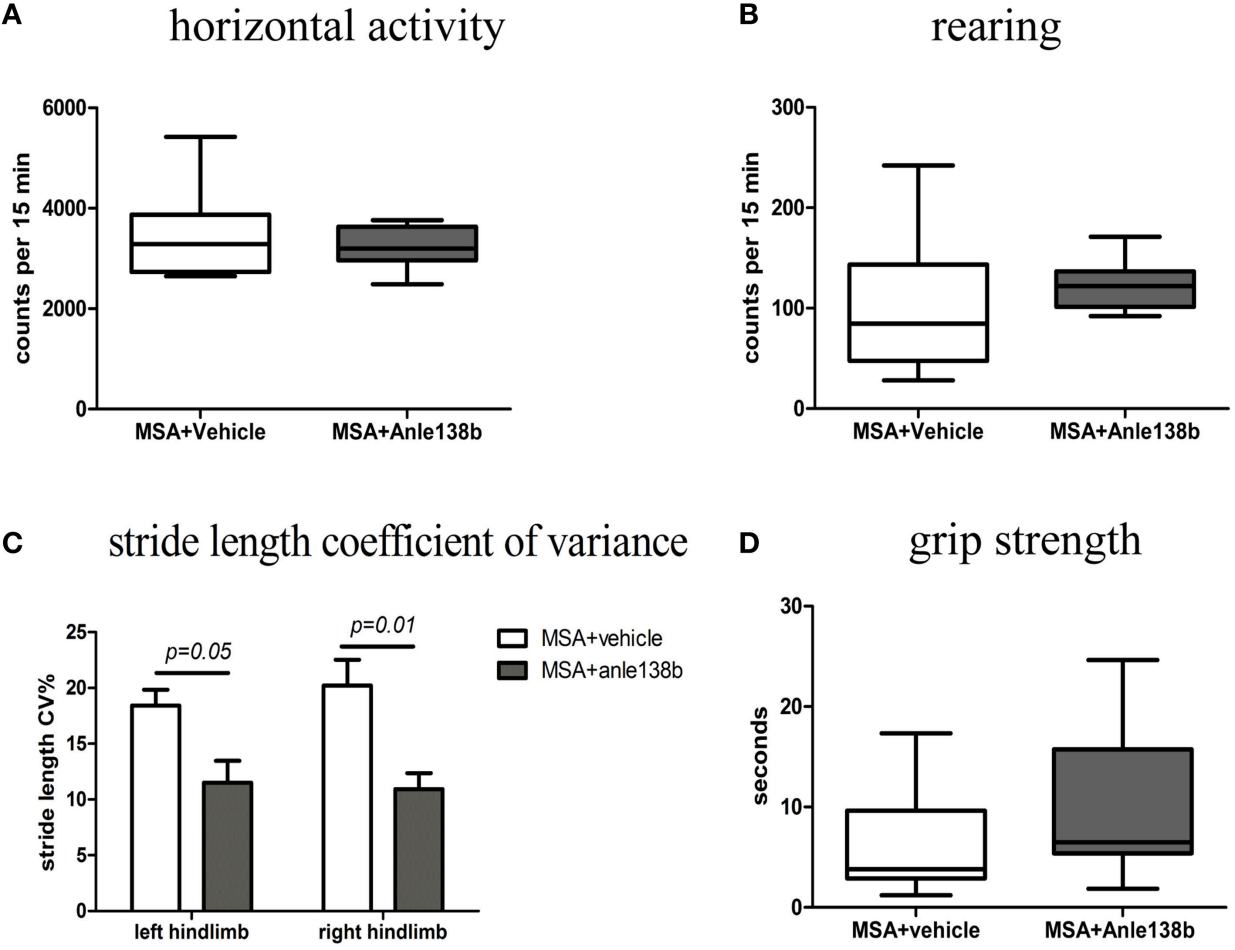
**Effects of anle138b treatment on motor behavior**. **(A)** Horizontal open-field activity showed no significant differences comparing vehicle- and anle138b-treated PLP-α-syn mice. **(B)** Vertical activity (rearing) in an open-field arena did not improve significantly upon anle138b administration as compared to vehicle treatment in PLP-α-syn mice. **(C)** Mice treated with anle138b showed a decreased variability of the percentage regarding the stride length coefficient of variation (CV) in both hindlimbs compared to the vehicle group using stride length analysis. **(D)** PLP-α-syn mice treated with anle138b revealed a slight improvement of grip strength compared to vehicle-treated mice, yet no statistical significance was reached. Data are presented as mean ± SEM. PLP-α-syn mice treated with vehicle *n* = 10, treated with anle138b *n* = 8. Data were analyzed by two-way ANOVA with *post-hoc* Bonferroni's test in **(C)**. Unpaired *t*-test was used for the comparison of the two groups **(A, B, D)**.

**Figure 3:**
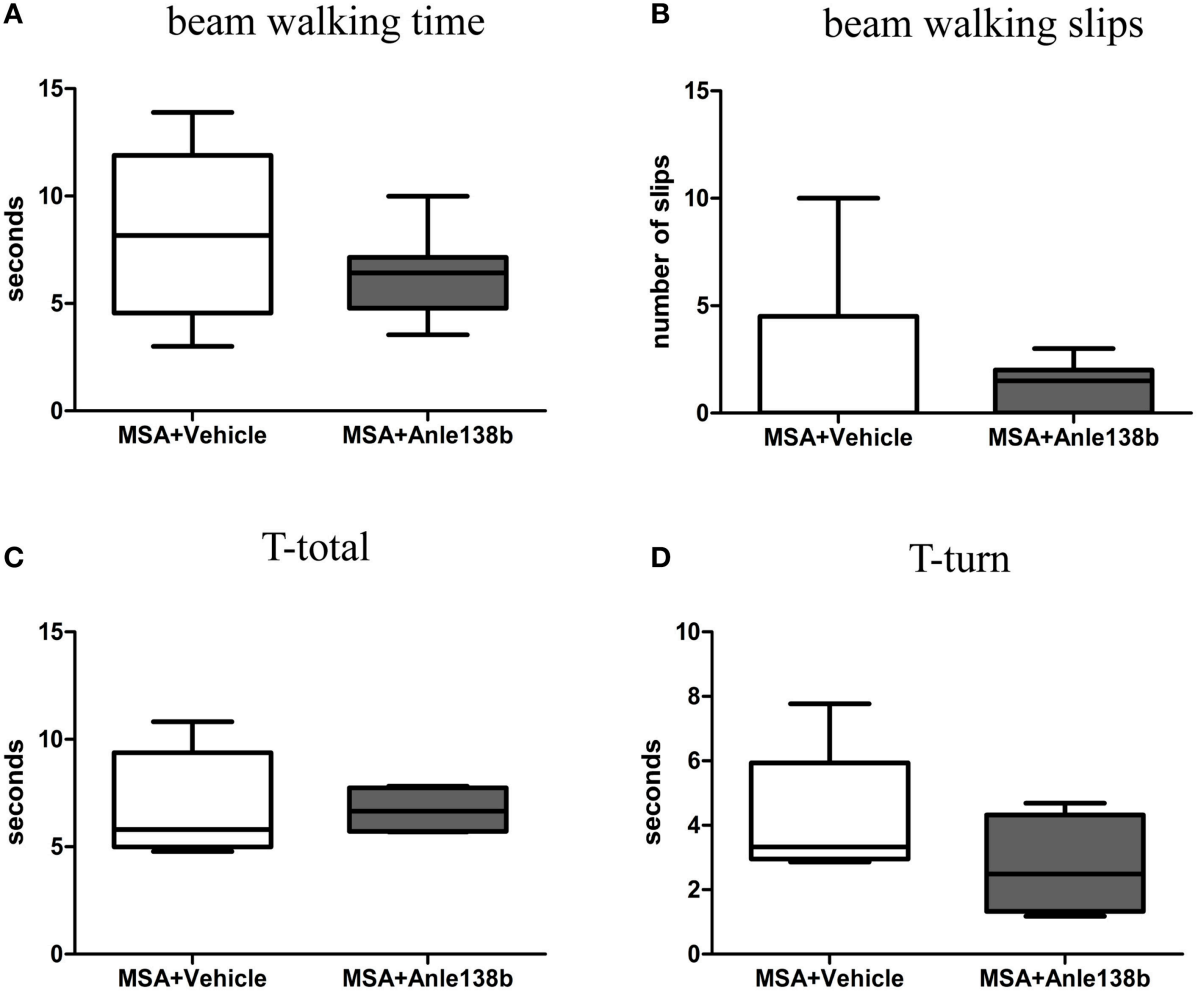
**Effects of anle138b treatment on motor behavior**. Beam walking performance showed a numeric improvement regarding beam walking time **(A)** and slips **(B)** in anle138b-treated mice compared to vehicle-treated mice. Furthermore, mice treated with anle138b presented with mild ameliorated behavior in the pole test when measuring T-total **(C)** and T-turn **(D)** time compared to the vehicle group. However, no statistical significance was reached in any of these tests. Data are presented as mean ± SEM. PLP-α-syn mice treated with vehicle *n* = 10, treated with anle138b *n* = 8. Unpaired *t*-test was used for the comparison of the two groups.

General locomotor activity in the vertical (rearing) and horizontal plane in an open field arena showed no significant differences between vehicle- and anle138b-treated PLP-α-syn mice (Figures 2A,B). However, analysis of the stride length revealed a significantly reduced variability of the stride length coefficient of variation (CV) in hindlimbs of anle138b treated mice compared to the vehicle group (Figure 2C). Assessment of the grip strength revealed that anle138b-treated PLP-α-syn mice were able to perform the test for a while longer compared to vehicle-treated mice. Yet, no statistical significance was reached regarding this test (Figure 2D). Furthermore, we found a numeric reduction of beam walking time (Figure 3A) and slips (Figure 3B) in the beam walking test, as well as a decreased T-total (Figure 3C) and T-turn (Figure 3D) time in the pole test when comparing anle138b with vehicle-treated animals, but none of these parameters reached statistically significant change due to the treatment with anle138b.

### Anle138b does not rescue dopaminergic and purkinje neurons in 3-NP-treated PLP-α-syn mice

**Figure 4:**
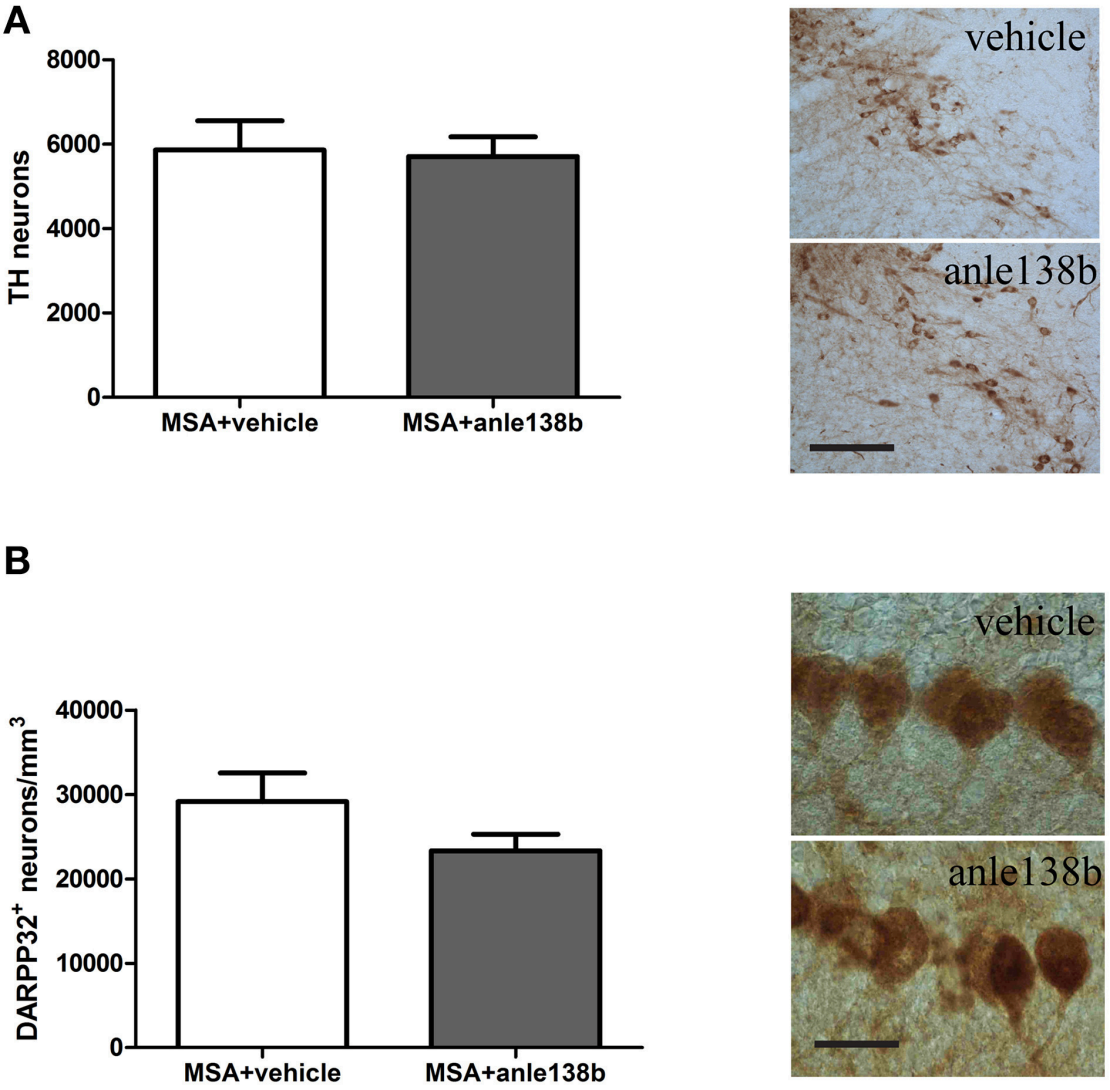
**Impact of anle138b on dopaminergic and Purkinje neuronal cells**. A loss of dopaminergic neurons **(A)** and Purkinje cells **(B)** was induced with the administration of 3-NP to PLP-α-syn mice compared to neuronal numbers in PLP-α-syn mice as previously shown (Stefanova et al., 2005). Administration of anle138b did not lead to the rescue of dopaminergic neurons (*scale bar* 100 μm) **(A)** as well as Purkinje neurons (*scale bar* 25 μm) **(B)**. Data are presented as mean ± SEM. PLP-α-syn mice treated with vehicle *n* = 10, treated with anle138b *n* = 8. Unpaired *t*-test was used for the comparison of the two groups.

Loss of TH-immunoreactive neurons in SNc was induced by systemic treatment with 3-NP in PLP-α-syn mice as shown by the low total number of stereologically estimated TH-positive neurons (Figure 4A) compared to TH numbers in PLP-α-syn mice as previously shown (Stefanova et al., 2005). Administration of the aggregation inhibitor anle138b did not show protection of dopaminergic neurons in SNc compared to vehicle treated mice (Figure 4A). Furthermore, a reduction of Purkinje cells was induced by the 3-NP treatment as indicated by the low number of Purkinje cells per mm^3^ (Figure 4B) compared to Purkinje cell numbers in PLP-α-syn mice as previously shown (Stefanova et al., 2005). Anle138b-treated mice showed no significant change regarding the numbers of Purkinje neurons as compared to vehicle-treated mice (Figure 4B).

### PLP-α-syn mice treated with Anle138b do not present with a reduced number of α-syn-positive inclusions

**Figure 5:**
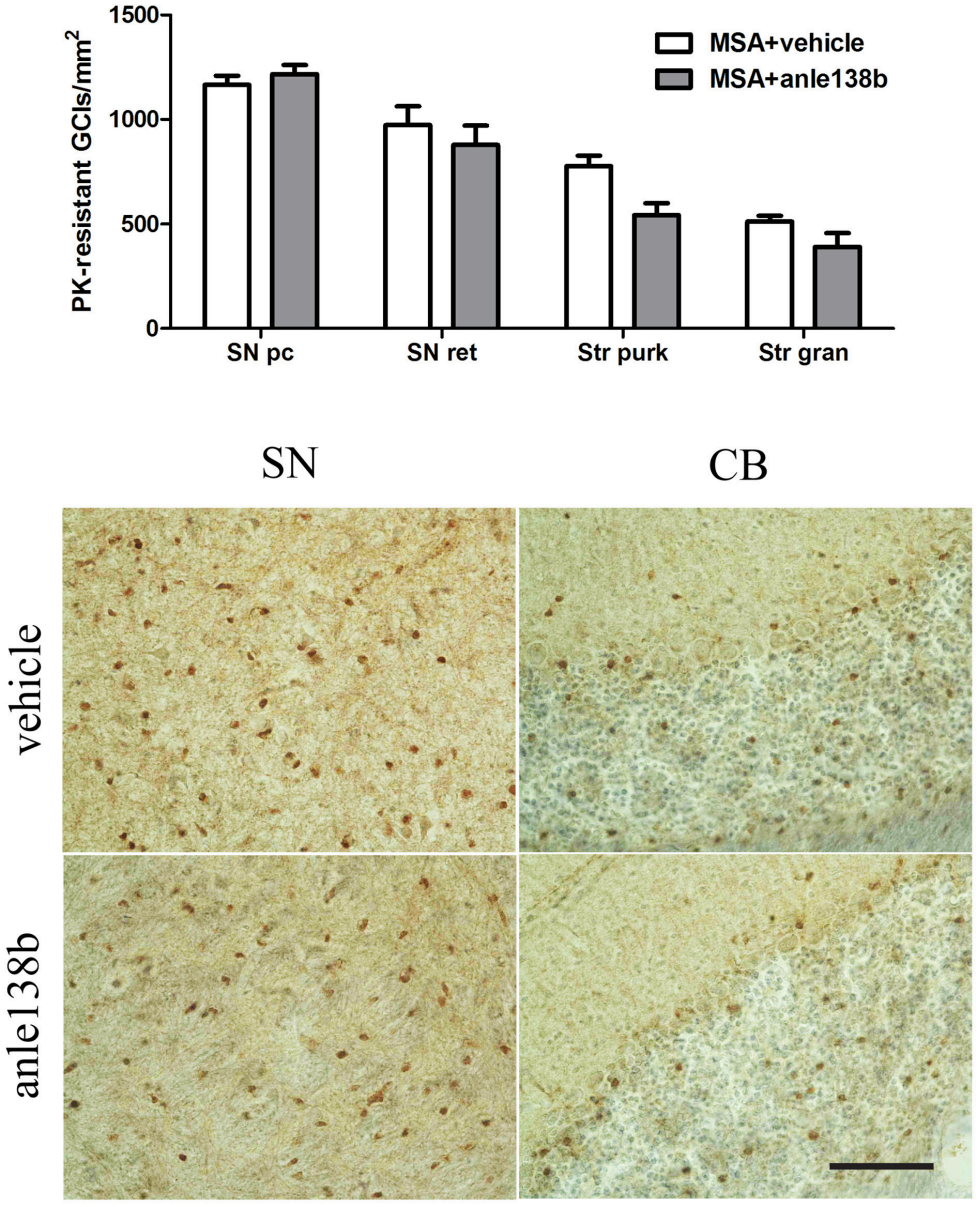
**Anle138b showed no effect on numbers of PK-resistant GCIs in PLP-α-syn mice**. No reduction in the number of PK-resistant GCIs was observed after administration of anle138b in 3-NP-treated PLP-α-syn mice compared to the vehicle group. Neither in SN (SNc, SNr) nor in the CB [Stratum granulosum (Str gran) and purkinjense (Str purk)] a reduced number of PK-resistant GCIs was noted as a result of anle 138b treatment. Region-specific differences were found with a significant lower number of PK-resistant GCIs in the CB compared to the SN [*F*_(3, 1)_= 2.25, *p* < 0.0001] but these remained independent of the therapy. *Scale bar* 30 μm. Data are presented as mean ± SEM. PLP-α-syn mice treated with vehicle *n* = 10, treated with anle138b *n* = 8. Data were analyzed by two-way ANOVA with *post-hoc* Bonferroni's test.

**Figure 6:**
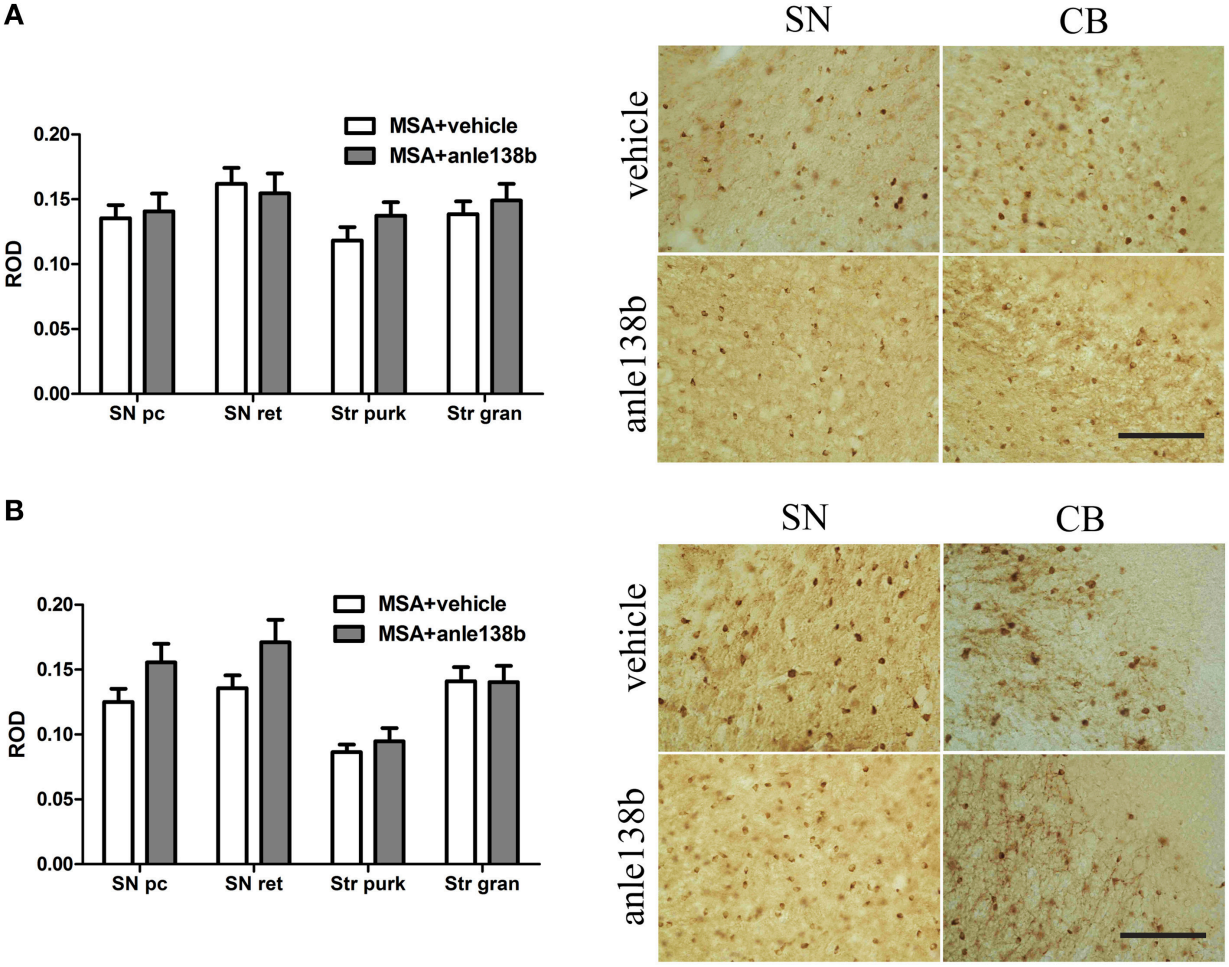
**Effects of anle138b on nitrated and phosphorylated α-syn species in PLP-α-syn mice**. The relative optical density (ROD) of nitrated α-syn measured in the SN (SNc, SNr) and CB [Stratum granulosum (Str gran) and purkinjense (Str purk)] did not reveal any significant differences between anle138b-compared to vehicle-treated PLP-α-syn mice. Moreover no region-specific differences were found [*F*_(3, 1)_ = 0.6781, *p* = 0.0841] **(A)**. Furthermore, no differences between the two treatment groups were found regarding the ROD of phosphorylated α-syn-species in SN and CB. ROD values in stratum purkinjense were lower compared to the other regions investigated [*F*_(3, 1)_ = 5.005, *p* < 0.0001] but still independent of the anle138b treatment **(B)**. *Scale bar* 25μm. Data are presented as mean ± SEM. PLP-α-syn mice treated with vehicle *n* = 10, treated with anle138b *n* = 8. Data were analyzed by two-way ANOVA with *post-hoc* Bonferroni's test.

Oral treatment with the compound anle138b had no significant effect on the number of PK-resistant GCIs in any of the regions studied as compared to vehicle-treated mice. Independent of the anle138b treatment, region-specific differences were detectable between SN (SNc and SNr) and cerebellum [CB, stratum granulosum and stratum pukinjense; *F*_(3, 1)_ = 2.25, *p* < 0.0001] revealing a significantly lower number of PK-resistant GCIs in CB compared to SN (Figure 5). In parallel, no significant differences in the optical density of nα-syn and pα-syn was determined between vehicle- and anle138b-treated mice in SN and CB (Figure 6). No region-specific differences regarding the optical density of nα-syn were found [*F*_(3, 1)_ = 0.6781, *p* = 0.0841; Figure 6A], but the optical density of pα-syn revealed weaker signal in Stratum purkinjense compared to SN [*F*_(3, 1)_ = 5.005, *p* < 0.0001; Figure 6B].

## Discussion

As MSA is a fatal primary oligodendrogliopathy associated with GCIs and selective multisystem neurodegeneration novel therapeutic drugs that attenuate brain pathology are of great importance. In the current study we investigated for the first time the effect of the novel aggregation inhibitor anle138b on behavioral outcome, neurodegeneration and α-syn aggregates in MSA mice exposed to mitochondrial inhibition. We observed improvement of stride length variability in anle138b treated MSA mice. However, there were no significant effects on loss of dopaminergic neurons or Purkinje cells and on GCI density in SN and CB.

Promising results exist regarding the reduction of neurodegeneration and pathological deposition of α-syn in different PD mouse models (rotenone, MPTP, and neuronal overexpression of α-syn) upon anle138b administration (Wagner et al., 2013; Levin et al., 2014). After oral administration, anle138b was shown to have a good bioavailability and blood-brain barrier penetration without toxicity, to ameliorate motor dysfunction, to block dopaminergic cell death, to interfere directly with the pathological aggregation of α-syn *in vitro* and *in vivo* as well as to prolong survival even in late-stage treatment (Wagner et al., 2013). In the MSA model applied in the current study, IP administration of 3-NP in transgenic PLP-α-syn mice leads to profound behavioral motor impairment as indicated by the motor score, open-field behavior, pole test performance, and stride length (Stefanova et al., 2005). In agreement with the findings of Wagner et al. (2013) we demonstrated a mild motor improvement in the MSA mouse including significantly reduced stride length variability linked to anle138b treatment. Additionally, we observed a numeric but not significant amelioration of beam walking and pole test performance suggesting a tendency to an improved motor function and coordination. Horizontal and rearing open-field behavior is impaired by the effect of 3-NP treatment as shown before (Stefanova et al., 2005), yet anle138b could not ameliorate open field behavior in this experimental setup. We did not find a significant improvement in the motor score of anle138b-treated compared to vehicle-treated mice. As the motor score estimates a variety of changes regarding the motor phenotype of mice, including hindlimb clasping, general locomotor activity, hindlimb dystonia, truncal dystonia, and postural challenge response (Fernagut et al., 2002), the scale might be too broad to pick up mild improvement in the anle138b group compared to the vehicle group. The mild improvement in motor deficits of MSA mice treated with anle138b is underlined by the neuropathological findings. As expected, treatment with 3-NP caused a profound loss of TH-immunoreactive neurons in SN and Purkinje cells in the CB in PLP-α-syn mice as shown previously (Stefanova et al., 2005). In contrast to the rescue of dopaminergic neurons in the SN in a sub-acute MPTP PD model treated with anle138b (Wagner et al., 2013), we did not observe a rescue of dopaminergic neurons, or Purkinje neurons upon treatment with the aggregation inhibitor anle138b in the 3-NP challenged PLP-α-syn mouse model. Furthermore, the amount of pathological α-syn aggregates in the brain of a PD mouse model overexpressing α-syn under a neuronal promoter was strongly reduced by anle138b (Wagner et al., 2013). In our study, we were not able to detect a decrease of α-syn aggregates in SN and CB in anle138b treated PLP-α-syn mice. The amount of pα-syn and nα-syn did not change when comparing the anle138b and the vehicle group. However, at this stage we cannot exclude effects of anle138b treatment on α-syn oligomers that has not been assessed in this experiment.

One reason for the limited improvement in the current study compared to earlier studies, could be that the compound anle138b was either tested in synucleinopathy models created with toxins OR neuronal overexpression of α-syn, but never in a dual-hit mouse model (Wagner et al., 2013). In the mouse model used in this study, the overexpression of α-syn in oligodendrocytes combined with 3-NP-induced oxidative stress results in severe full-blown MSA-like neuropathology (Stefanova et al., 2005) which might necessitate the application of higher doses of anle138b to counteract. Furthermore, in the previously reported PD models, rotenone and MPTP act via effects on α-syn aggregation in neurons to induce dopaminergic loss that can be rescued by anle138b (Wagner et al., 2013; Levin et al., 2014), while it may be speculated that 3-NP and oligodendroglial α-syn aggregation in the MSA model trigger neuronal loss by different mechanisms (not involving neuronal α-syn accumulation during 3-NP challenge) which may have led to the failure of anle138b to protect the degenerating neurons in this mouse model.

Of note, we were not able to identify changes in α-syn inclusion bodies in oligodendrocytes in the MSA model after 1 month of treatment with anle138b, although the drug has been shown previously to counteract α-syn aggregation. As already mentioned we cannot exclude that the duration and the dose of the therapy in this experiment have been insufficient to provide measurable changes in the GCI density and further studies will be needed to address this issue as well as potential effects on α-syn oligomers. Furthermore, in a recent study, 3-NP was shown to impair the autophagic flux in oligodendroglial cells treated with exogenously added α-syn *in vitro*, inducing increased number and size of α-syn aggregates (Pukass et al., 2015). The unchanged amount of α-syn aggregates in our PLP-α-syn mouse after treatment with anle138b might indicate a defective autophagic pathway after 3-NP intoxication in oligodendroglial cells in the mouse model of the present study. Therefore, investigating the therapeutic properties of anle138b in the PLP-α-syn mouse model without additional oxidative stress is likely to represent a more relevant approach for targeting α-syn aggregation in MSA (Stefanova and Wenning, 2015).

Comparing human PD to MSA, the latter seems to be the more progressive neurodegenerative disease with a fatal outcome after ~9 years whereas PD patients have a better prognosis (Schrag et al., 2008; Lipp et al., 2009). In PD α-syn-positive inclusions occur predominantly in neurons whereas in MSA oligodendroglial α-syn inclusions are more common (Bruck et al., 2015). The mechanism of the development of Lewy bodies (LBs) in PD and GCIs in MSA has not been resolved by now. Differences in the formation, distribution and impact of GCIs in MSA vs. LBs in PD (Del Tredici and Braak, 2015) cannot be excluded to contribute to potential differences in the effects of anle138b. The differences in pathology (oligodendroglial vs. neuronal inclusions) indicate diverging molecular pathways and latest findings show that different α-syn conformations (oligomers, ribbons, fibrils; Peelaerts et al., 2015) or α-syn strains (Prusiner et al., 2015; Woerman et al., 2015) might exist and act differently to induce different disease patterns, suggesting that a compound effective in PD may be less efficient in the same dose in MSA. We cannot exclude, however, that the oxidative stress induced by 3-NP treatment dominates the phenotype of the mice and since anle138b does not have antioxidant effects it cannot rescue this aspect. Indeed, nitration and phosphorylation of α-syn were not changed by anle138b as expected.

In conclusion, we here report for the first time the effects of the new aggregation inhibitor anle138b in a MSA mouse model combining oligodendroglial α-syn overexpression with 3-NP-induced oxidative stress. Taken together, the data suggest that anle138b induced some improvement of motor impairment supporting further development of the compound for MSA. However, no significant protection of neuronal cells or reduction of oligodendroglial α-syn aggregation was found. Advanced MSA pathology reproduced in the dual-hit mouse model might have been too severe to permit significant neuroprotection with anle138b treatment in the applied dose and duration. Furthermore, interference between oxidative stress and clearance mechanisms of α-syn may have masked the effects of anle138b treatment on oligodendroglial α-syn aggregation in the double-hit model of MSA. Future studies will need to address all these issues to resolve the efficacy of anle138b for the therapy of MSA.

## Author contributions

LF—acquisition, analysis, and interpretation of data; drafting and revising the manuscript; final approval of the submission; agreement to be accountable for all aspects of the work in ensuring that questions related to the accuracy or integrity of any part of the work are appropriately investigated and resolved. DK—acquisition of data; revising the manuscript; final approval of the submission; agreement to be accountable for all aspects of the work in ensuring that questions related to the accuracy or integrity of any part of the work are appropriately investigated and resolved. JL, SR, AL, and CG—interpretation of data; revising the manuscript; final approval of the submission; agreement to be accountable for all aspects of the work in ensuring that questions related to the accuracy or integrity of any part of the work are appropriately investigated and resolved. AG, GW, and NS—conception and design; interpretation of data; revising the manuscript; final approval of the submission; agreement to be accountable for all aspects of the work in ensuring that questions related to the accuracy or integrity of any part of the work are appropriately investigated and resolved. All authors read and approved the final manuscript.

### Conflict of interest statement

AG and CG are co-founders of MODAG. AL is partly employed by MODAG. Remaining co-authors declare that the research was conducted in the absence of any commercial or financial relationships that could be construed as a potential conflict of interest.
